# Environmental DNA: A New Low-Cost Monitoring Tool for Pathogens in Salmonid Aquaculture

**DOI:** 10.3389/fmicb.2018.03009

**Published:** 2018-12-07

**Authors:** Lucy Peters, Sofie Spatharis, Maria Augusta Dario, Toni Dwyer, Inaki J. T. Roca, Anna Kintner, Øyvind Kanstad-Hanssen, Martin S. Llewellyn, Kim Praebel

**Affiliations:** ^1^Institute of Evolutionary Biology, University of Edinburgh, Edinburgh, United Kingdom; ^2^Institute of Biodiversity, Animal Health and Comparative Medicine, University of Glasgow, Glasgow, United Kingdom; ^3^School of Life Sciences, University of Glasgow, Glasgow, United Kingdom; ^4^Laboratório de Biologia de Tripanosomatídeos, Instituto Oswaldo Cruz (IOC/Fiocruz), Rio de Janeiro, Brazil; ^5^Norwegian College of Fishery Science, University of Tromsø, Tromsø, Norway; ^6^Ferskvannsbiologen, Ltd., Lødingen, Norway

**Keywords:** *Pseudo-nitzschia seriata*, *Prymnesium parvum*, *Lepeophtheirus salmonis*, *Paramoeba perurans*, environmental DNA (eDNA), aquaculture

## Abstract

Environmental DNA (eDNA) metabarcoding is a relatively new monitoring tool featuring in an increasing number of applications such as the facilitation of the accurate and cost effective detection of species in environmental samples. eDNA monitoring is likely to have a major impact on the ability of salmonid aquaculture industry producers and their regulators to detect the presence and abundance of pathogens and other biological threats in the surrounding environment. However, for eDNA metabarcoding to develop into a useful bio-monitoring tool it is necessary to (a) validate that sequence datasets derived from amplification of metabarcoding markers reflect the true species’ identity, (b) test the sensitivity under different abundance levels and environmental noise and (c) establish a low-cost sequencing method to enable the bulk processing of field samples. In this study, we employed an elaborate experimental design whereby different combinations of five biological agents were crossed at three abundance levels and exposed to sterile pre-filtered and unfiltered seawater, prior to coarse filtering and then eDNA ultrafiltration of the resultant material. We then benchmarked the low-cost, scalable, Ion Torrent sequencing method against the current gold-standard Illumina platform for eDNA surveys in aquaculture. Based on amplicon-seq of the 18S SSU rDNA v9 region, we were able to identify two parasites (*Lepeophtheirus salmonis* and *Paramoeba perurans)* to species level, whereas the microalgae species *Prymnesium parvum, Pseudo-nitzschia seriata*, and *P. delicatissima* could be assigned correctly only to the genus level. Illumina and Ion Torrent provided near identical results in terms of community composition in our samples, whereas Ion Torrent was more sensitive in detecting species richness when the medium was unfiltered seawater. Both methods were able to reflect the difference in relative abundance between treatments in 4 out of 5 species when samples were exposed to the unfiltered seawater, despite the significant amount of background noise from both bacteria and eukaryotes. Our findings indicate that eDNA metabarcoding offers significant potential in the monitoring of species harmful to aquaculture and for this purpose, the low-cost Ion Torrent sequencing is as accurate as Illumina in determining differences in their relative abundance between samples.

## Introduction

The salmonid aquaculture industry is undergoing explosive growth globally. However, the industry is beset by parasitic disease and is often the subject to mass mortalities of farmed fish due to toxin-producing Harmful Algal Blooms (HABs) (Smayda, 2006; Hinder et al., 2011). Economic losses associated with certain agents, as for example sea lice, accounts for up to £ 470 M/year for major producers like Norway (Liu and Bjelland, 2014). The presence and abundance thresholds of these potentially damaging organisms in the environment and around aquaculture sites must be closely monitored. Traditional microscopy methods for algal and copepod larval species identification are time-consuming, demand expertise and are not always accurate when abundances are low or when cryptic species are involved. Similarly, parasite counts on the fish themselves are both time consuming and impose significant handling stress.

Environmental DNA (eDNA) analysis is an emerging molecular approach for species identification from samples containing cellular DNA and extracellular DNA sloughed off all living organisms (Bohmann et al., 2014). eDNA analysis has been successfully employed to detect and monitor eukaryotic micro- and macrobial communities and populations (Ficetola et al., 2008; Thomsen et al., 2012; Zamor et al., 2012) and is a useful tool for early monitoring systems as it allows for more accurate and standardized detection of species that are cryptic, inaccessible (Thomsen et al., 2012) and of low abundance (Ficetola et al., 2008). There have been several studies that have validated eDNA as a monitoring tool (Young et al., 2008; Olson et al., 2012; Zamor et al., 2012; Mahon et al., 2013; Wood et al., 2013) with some recent advances toward its application for pathogen detection in freshwater aquaculture (Gomes et al., 2017). However, before it can be considered as a systematic bio-monitoring tool it is necessary to find a cost-effective analytical approach to allow rapid processing of environmental samples on a day-to-day basis. Furthermore, validation is required to establish the relationship between eDNA genotype data and biological abundance (e.g., Nathan et al., 2014).

Next generation sequencing (NGS) methods are increasingly being used to identify aquatic organisms from eDNA samples (Eiler et al., 2013; Yu et al., 2015). Metabarcoding – the use of universal primers to amplify DNA from many different organisms within one sample – is the technique most frequently deployed in the context of NGS (Taberlet et al., 2012). Multiplex of samples via the inclusion of molecular identifier tags allows for parallel processing of multiple samples both during the sequencing run as well as during the downstream bioinformatic analysis using a technique termed amplicon-seq (Wood et al., 2013). Two commonly used NGS methods are Ion Torrent and Illumina MiSeq – the efficiency of which in detecting organisms from community samples has been compared in multiple studies mostly focused on bacterial diversity (e.g., Salipante et al., 2014; Clooney et al., 2016; Lahens et al., 2017). Several hundred samples can be multiplexed on a single Illumina MiSeq run, but the cost per run is relatively high. The Ion Torrent platform offers scalable sequencing runs, which provides more flexibility for running low numbers of samples (20–40 per chip) on a regular basis. Most of comparative studies suggest that Illumina and Ion Torrent have similar capacities to detect changes in biology from a treatment/control experiment, however, Ion Torrent seems to be more prone to error due to organism-related biases that lead to under-representation of certain species (Salipante et al., 2014). This error has been attributed to the premature truncation of sequences during semiconductor sequencing (Salipante et al., 2014).

Although detection of pathogens would be an important step forward for the aquaculture industry, it is also important to be able to assess whether the abundances of target pathogenic organisms have exceeded established thresholds regarding food safety or water quality^1^. Species quantification remains a holy grail for eDNA studies and several authors report progress toward this goal in aquatic organisms (Nathan et al., 2014), with more recent studies incorporating models of DNA shedding and degradation (Sassoubre et al., 2016). Absolute individual-level quantification is complicated in comparisons of metazoans and unicellular species where biomass, instead of count data, is likely to show better correlation with DNA quantity (Sassoubre et al., 2016). More recently, data processing techniques such as normalization of read data have provided a better representation of absolute abundances across experimental treatments (Weber and Pawlowski, 2013).

In this study, our aim was to establish whether eukaryotic aquaculture pathogens and harmful algae can be reliably detected and identified using a universal metabarcoding approach. Specifically, our objectives were to (1) test whether multiple pathogens can be detected simultaneously against a background of biological “noise” from the zooplankton, (2) establish whether artificially generated differences in relative proportion/abundance of the pathogens between samples can be detected, and (3) explore the power of different sequencing methodologies to generate the data. To achieve these aims we used an elaborate experimental design involving cross treatments of the target organisms at three abundance levels, exposed to background noise influence (i.e., unfiltered sea-water) versus sterile pre-filtered sea-water. We then benchmarked Illumina MiSeq and Ion Torrent techniques to deep sequence the ribosomal 18rRNA marker gene of these samples.

## Materials and Methods

### Choice of Pathogen

Five major protect pathogens and risk agents were selected: *Lepeophtheirus salmonis* and *Paramoeba perurans* as well as the algal risk agents *Prymnesium parvum* (identified morphologically in University of the Aegean), *Pseudo-nitzschia seriata* (identified by N. Lundholm, personal communication), and *P. delicatissima* (CCAP culture). *L. salmonis* is one of the most important and widespread, affecting farmed Atlantic salmon in Norway, Ireland, and the United Kingdom (Torrissen et al., 2013; Ellis et al., 2016). *P. perurans* is the causative agent of amoebic gill disease, which is a major source of commercial loss for aquaculture in Tasmania, but also affects industries in both North and South America as well as in Europe (Young et al., 2008). The haptophyte *P. parvum*, produces compounds known as prymnesins causing severe toxic effects by affecting plasma membrane integrity of gills (Manning and La Claire, 2010). *P. parvum* blooms resulted in extensive mortality of farmed salmon in Scotland and Norway in the past (Smayda, 2006). *Pseudo-nitzschia* produce the neurotoxin Domoic acid (DA), that causes Amnesic Shellfish Poisoning (ASP) symptoms in humans (Hinder et al., 2011) when it bioaccumulates in the tissue of bivalves.

### Incubations and Filtering

**FIGURE 1:**
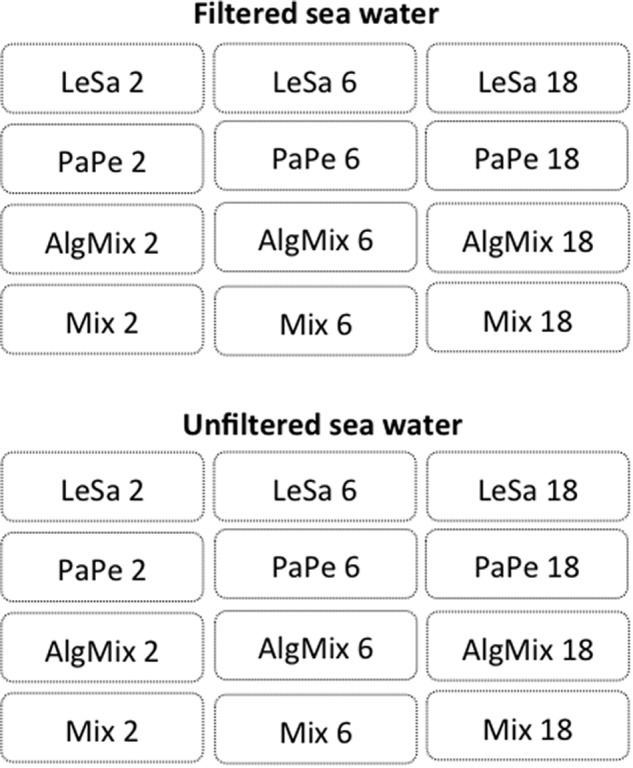
Experimental design aiming to test the efficiency of two sequencing methods, namely Illumina MiSeq and IonTorrent, in reflecting the species composition and abundance in different incubations. Each incubation contained four aquaculture related risk agents either in isolation or combined: *Lepeophtheirus salmonis* (LeSa), *Paramoeba perurans* (PaPe), algal mix (AlgMix) containing *Prymnesium parvum, Pseudonitzschia delicaticima, and P. seriata*, and Mix containing all the species together. Each of these incubations was performed at three abundance levels representing triplings of the initial abundance (i.e., 2, 6, and 18). The experiment aimed to control for the effect of background noise in these treatments thus we deployed these using both filtered and unfiltered seawater medium.

We incubated cultures of the different pathogens in four different groups: Group 1 containing *L. salmonis*, Group 2 containing *P. perurans*, Group 3 containing the combination of the three algae (*P. parvum, P. seriata*, and *P. delicatissima*) and group 4 containing a mix of all the species. Each of the four groups was further divided into three treatment regimens of the following abundances: two, six and eighteen, in the case of the salmon louse referring to number of individuals (females without egg strings), and to cell counts for the amoeba and algal species. The densities of the cultures of amoeba and algal species were determined by microscopy and dilution series in a Neubauer chamber. As a negative control, a blank baseline treatment was set up for each of the two different days of the incubations. All incubations consisted of 2.0 L of two different media; sterile filtered (0.22 μm) seawater and unfiltered seawater. In this way, the strength of the relationship between known abundances and sequence read numbers could be tested both for the baseline level of PCR amplification only, as simulated by the filtered medium, and in the context of the natural environment, replicated by the unfiltered medium. Both the unfiltered and filtered seawater were obtained from internal pipes of the marine laboratory of the Norwegian College of Fishery Science, UiT the Arctic University of Norway. Our experimental design generated 72 samples (2 medium types: filtered/unfiltered × 12 treatments × 3 replicates) (see Figure 1 for experimental design). For each medium type, we also used 2 blanks replicated thrice (12 samples in total), resulting in a total of 84 samples. All incubations were performed at 3.8°C for 24 h, before 0.5 L were filtered to collect material for DNA extraction and sequencing through a 0.22 μm sterivex filter unit (EMD Millipore, Darmstadt, Germany). Samples for all incubations were obtained in triplicate.

### DNA Extraction, PCR, and Sequencing

Total DNA was extracted using the DNeasy Blood and Tissue kit (Qiagen, Hilden, Germany) following the manufacturer’s instructions with the minor adjustments. Briefly, 500 μl instead of 200 μl digestion buffer ATL/proteinase K was added directly to each Sterivex filter and incubated over night with continues rotation. The buffer, containing the eDNA, was then spun out of the filters into a 2 ml Eppendorf tube at 1700 × *g* for 3 min. Each sample, was then added an equal volume as the eluate of the lysis buffer AL and 100% ethanol and vortexed. The mixture was transferred to a spin column and the manufactures protocol followed until the elution step where 75 μl EB was used instead of 200 μl. All handling of samples, from sampling water to extraction of eDNA and handling of extracts, was performed under strict clean conditions at designated clean labs for eDNA work at the Norwegian College of Fishery Science.

Sequencing libraries were generated using two rounds of PCR amplification. For the initial round of amplification, each PCR was conducted in a 25 μl reaction volume containing 10ng of template DNA, 0.5 μM of each primer and 12.5 units of Q5 Hot Start High-Fidelity 2X Master Mix (New England Biolabs: Ipswich, MA, United States). The following primers were used in the PCR reactions to amplify the 18S V9 region (Hadziavdic et al., 2014): forward primer 1391_F (5′-GTACACACCGCCCGTC-3′) and reverse primer 1560_R (5′-TGATCCTTCTGCAGGTTCACCTAC-3′) with the following conditions: one initial cycle of 15 min at 95°C; 35 cycles of 45 s at 95°C, 45 s at 58°C, and 60 s at 72°C; and one final cycle of 10 min at 72°C. Multiplex identifier tags were added to the first round PCR products by a second, five cycle, PCR reaction (45 s at 95°C, 45 s at 58°C, and 60 s at 72°C). The PCR profile of the fragment amplification for Ion Torrent sequencing only differed from this protocol in the duration of the different temperature phases within the PCR cycle, which were 30 s for each of the three phases. The PCR products were then run on a 2% agarose gel for quality control using SYBR safe (Thermo Fisher Scientific, Waltham, MA, United States) as an in-gel stain at x1 concentration and DNA bands were then visualized for inspection under UV light.

Illumina MiSeq paired-end sequencing of the 18S V9 region was carried out at the Glasgow Polyomics lab (Glasgow, Scotland, United Kingdom) using the MiSeq reagent kit (600 cycle) (Illumina, San Diego, CA, United States) and 2 × 300 bp sequencing. Ion Torrent sequencing of the amplicons was carried out in the Systems Biology Centre of the University of Plymouth (Plymouth, England, United Kingdom) on Ion 318v2 Chips, using the Ion PGM Sequencing 200 Kit v2 (Life Technologies Ltd., Carlsbad, CA, United States) for sequencing of up to 200 bp. For both sequencing procedures adapter sequences were trimmed automatically before the sequence data was exported as FASTQ files.

### Sequence Processing, OTU Clustering, and Taxonomic Assignment

Raw reads from the Illumina MiSeq and the Ion Torrent run were processed in the same manner using identical parameters, apart from some differences in pre-processing of the paired-end reads of the Illumina run. All raw reads, single for the Ion Torrent and both paired ends for the MiSeq run, were trimmed with Sickle version 1.33 (Joshi and Fass, 2011) using a quality threshold of 20 and a minimum length of 100 base pairs. For the paired ends, a file with singletons was created at this step, which was excluded from further analysis. The reads were further trimmed using FASTX-Toolkit version 0.0.14 (Hannon Lab) to a maximum length of 200 base pairs. All sequences were then aligned to a reference consisting of representative 18S sequences of all target organisms or a closely related species (GenBank accession numbers: AF208263.1 (*Lepeophtheirus salmonis*); KT989881.1 (*Paramoeba perurans*); KJ756812.1 (*Prymnesium parvum*); JF308618.1 *(Pseudo-nitzschia seriata*); EU478793.1 (*Pseudo-nitzschia delicatissima*) in an attempt to filter out non-18S reads. The alignment was carried out in bowtie2 version 2.2.6 (Langmead and Salzberg, 2012) using the low stringency local alignment option.

The matched reads then underwent further quality checking using PRINSEQ-lite version 0.20.4 (Schmieder and Edwards, 2011) to identify any formatting errors and remove read headers as well as convert the sequences into FASTA format to facilitate downstream processing. The paired-end MiSeq reads were further processed by merging the mate pairs into one sequence using Velvet version 1.2.09 (Zerbino and Birney, 2008) after reversing and complementing reads two. Merged MiSeq and single Ion Torrent reads were further processed using USEARCH version 8.1.1861 (Edgar, 2010): reads were scanned for unique sequences, sorted and finally clustered into operational taxonomic units (OTUs) using the UPARSE algorithm and a 97% identity threshold.

A table listing the OTUs and read frequency for every sample was constructed for each of the two sequencing methods. Taxonomic identity of the OTUs was assigned in Qiime version 1.9.1 (Caporaso et al., 2010) using the closed reference approach with the SILVA database release 128 (Quast et al., 2013) as a reference, which is the most comprehensive database for eukaryotic 18S sequences, and the BLAST algorithm for assignment. Multiple identical assignments for different OTUs were pooled together using Primer6 version 6.1.4 (Clarke and Gorley, 2006).

Sequence reads generated in this study have been submitted to the NCBI short-reads archive (SRA), accession number PRJNA505454.

### Sequencing Efficiency of MiSeq Versus Ion Torrent

Sequencing success of MiSeq sequencing compared to Ion Torrent sequencing was quantified by the percentage of raw reads retained after quality filtering as well as by total numbers of unique OTUs identified. Overall sequencing output and sample composition for both sequencing methods were further explored by determining absolute and relative read abundances using the phyloseq package version 1.18.0 (McMurdie and Holmes, 2013) in the statistical analysis software R version 3.3.0 (R Core Team, 2016). The effect of treatment, sequencing method and medium type on the number of OTUs and evenness index J was tested using General Linear Models of the form:

(1) $$\mathrm{OTU}\;\mathrm{richness}={\mathrm{Method}}_{i}+{\mathrm{Medium}}_{j}+{\mathrm{Treatment}}_{k}.$$

(2) $$\mathrm{Evenness}\;\mathrm{richness}={\mathrm{Method}}_{i}+{\mathrm{Medium}}_{j}+{\mathrm{Treatment}}_{k}.$$

Where: method is a categorical variable with two levels (i = Illumina MiSeq, Ion Torrent), Medium is a categorical variable with two levels (j = filtered, unfiltered), Treatment is a categorical variable with 12 levels (k = LeSa _2,6,18_, PaPe _3,6,18_, AlgMix _3,6,18_, Mix _3,6,18_).

### Sensitivity of MiSeq Versus Ion Torrent to Reflect Sample Composition

Potential differences between the two sequencing methods in reflecting OTU composition in the samples was explored using multivariate statistics. For this analysis the OTU data were first normalized to account for inter-sample variation in sequencing depth. Data normalization was conducted in the R package Deseq2 version 1.14.0 (Love et al., 2014) with which instead of rarefying, a variance stabilizing transformation was applied to the read numbers to convert the counts so that they are of homoscedastic distribution, after the recommendations of McMurdie and Holmes (2014). The normalized read samples were then analyzed within each method for pairwise similarity using the Bray-Curtis similarity index. The pairwise similarities between all samples were then visualized using Multidimensional Scaling Ordination (MDS).

### Sensitivity of MiSeq Versus Ion Torrent to Reflect Differences in Relative Abundances Between Treatments

The two sequencing methods of MiSeq and Ion Torrent were compared in their efficiency in reflecting the relative abundances of species within each medium type, i.e., filtered and unfiltered medium using a General Linear model of the form:

(3) $${\mathrm{Species}}^{\prime }\;\mathrm{OTU}\;\mathrm{reads}={\mathrm{Treatment}}_{k}.$$

Where k = 2, 6, and 18 abundance levels. The first order interaction between treatment and method and treatment and medium was also tested in order to check whether the effect of treatment depends on which method or which medium is being used. The relative (normalized) reads were used for this analysis and for the unfiltered samples we subtracted background concentrations of our target species that were found in the blanks from the abundances found in the treatments. No blank contained more than six reads from any one target species.

## Results

### Sequencing Efficiency and Taxonomic Assignment of MiSeq Versus Ion Torrent

The Illumina platform successfully sequenced 68 out of the 84 samples whilst Ion Torrent returned a slightly lower number (62 out of the 84 samples). The two sequencing approaches showed differences in their raw read numbers and overall sequence read quality. The Illumina MiSeq data consisted of fewer raw reads (6,115,810 paired reads) across all samples compared with the Ion Torrent output (9,350,400 single reads). After quality filtering of the raw reads, 93.7% of the MiSeq sequence pairs were kept compared to just 68.3% of the Ion Torrent raw reads. As a result the total number of reads that passed QC were similar for both sequencing methods. Samples from the *L. salmonis* incubation in unfiltered samples failed sequencing on both platforms, presumably due to problems with the first round PCR.

The OTUs of our algal species of interest were the most abundant reads amplified from the filtered samples containing the algal mix, and their identity was validated by a nucleotide BLAST search against the Genbank database on the NCBI website. As expected, both sequencing methods resulted in OTUs that could be assigned taxonomic identities to our target algae species with equal efficiency. However, some algal assignments were different to the known identity of the target organisms we introduced. The OTU that was assigned to *Prymnesium* was first mis-assigned as *P. nemathicium* using SILVA but later correctly identified as *Prymnesium parvum* by the top NCBI BLAST hit (100% sequence identity). As such this OTU was identified as *P. parvum* for downstream analysis. The validation of the *Pseudo-nitzschia* spp. identities was more ambiguous since our target species, *Pseudo-nitzschia seriata* and *P. delicatissima*, were assigned as *Pseudo-nitzschia australis* and *cuspidata* using SILVA and these taxonomic identities were confirmed by the top BLAST hits for these OTUs. *L. salmonis* and *P. perurans* were correctly assigned to species level with reference to both SILVA and NCBI BLAST.

**FIGURE 2:**
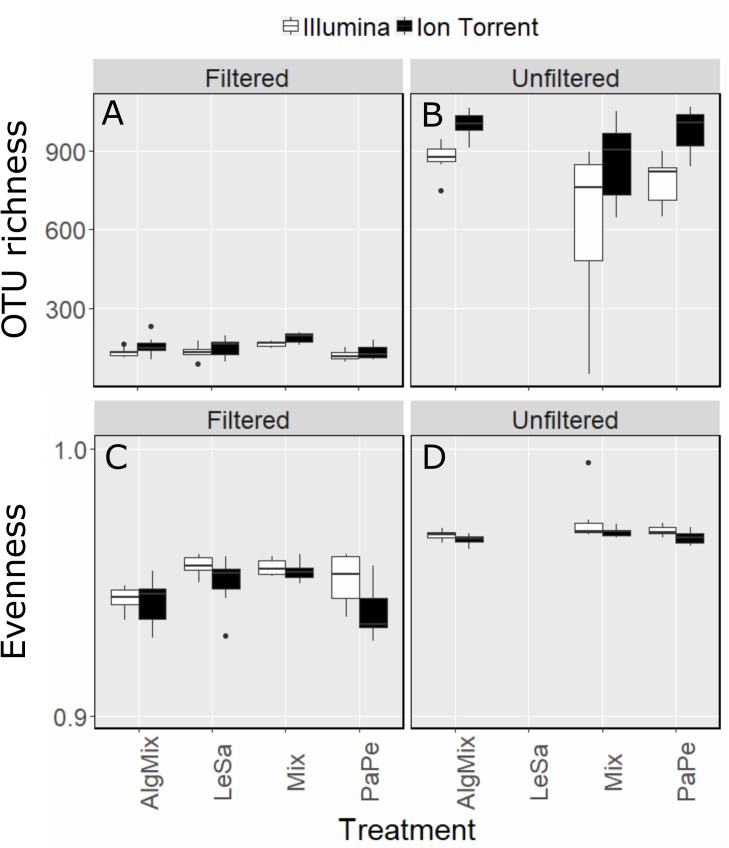
Comparison between Illumina MiSeq (green bars) and Ion Torrent sequencing (gray bars) in reflecting OTU richness **(A,B)** and evenness **(C,D)** across the four treatment levels: *Lepeophtheirus salmonis* (LeSa), *Paramoeba perurans* (PaPe), algal mix (AlgMix) containing the three algal species, and Mix containing all the five species together. Within each panel the two different methods are compared.

Once the OTU tables from each method were collapsed to avoid multiple identical taxonomic assignments, the Ion Torrent data appeared to capture slightly more diversity across all samples (2463 OTUs) compared to MiSeq (2277 OTUs). This was also observed on a per treatment basis (Figures 2A,B) as the effect of method was found significant (GLM, F-ratio = 13.5, *p* < 0.001) having accounted for medium type (pre-filtered versus unfiltered seawater). As expected the number of OTUs was much larger in the unfiltered medium type than the pre-filtered samples (GLM, F-ratio = 1329.8, *p* < 0.001). The effect of method depended on the medium type with the Illumina MiSeq performing significantly better than Ion Torrent in detecting more OTUs in the unfiltered sea water medium compared to the filtered one (GLM, F-ratio = 16.5, *p* < 0.001). Although sample evenness (i.e., how evenly reads are distributed across OTUs) was significantly lower across treatments (Figures 2C,D) for the Ion Torrent method (GLM, F-ratio = 17.2, *p* < 0.001) for both filtered and unfiltered medium (GLM, F-ratio = 1.3, *p* = 0.249), nevertheless the effect size was very small as in both methods evenness ranged between 0.93 and 0.98 (evenness can range from 0 to 1). Complete OTU tables can be found for each of the sequencing methods in the Supplementary Material.

### Sensitivity of Illumina MiSeq Versus Ion Torrent to Reflect Sample Composition

**FIGURE 3:**
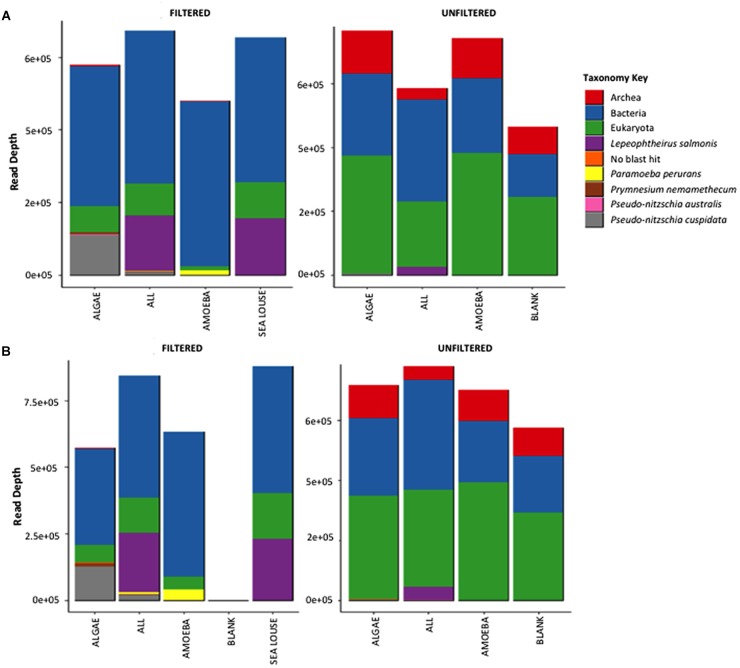
Absolute read numbers for samples grouped according to treatment and split for medium type. Color code identifies different taxonomic groups. **(A)** Represents the MiSeq data, **(B)** the data from the Ion Torrent run.

Both methods detected similar proportions of taxonomic groups considering the absolute read numbers across incubation treatments (Figure 3). Treatments containing unfiltered medium were more diverse in other taxonomic groups including bacteria, archaea and eukaryotes other than our target species. By comparison, in the filtered samples only bacteria were present apart from our target species. The Illumina Miseq and Ion Torrent methods were identical in depicting sample OTU composition as seen both by the relative contribution of taxa they detect (Figure 3) as well as the pairwise similarity between treatments (Supplementary Figure S1). Specifically, community composition in the unfiltered samples was clearly separated from the filtered samples using data from either sequencing method (Supplementary Figure S1). Also, samples from different treatments were grouped together at equal similarity within each method, showing more pronounced separation of treatments in the pre-filtered medium that the unfiltered one. The greater similarity observed in the unfiltered treatments was because of the interference of the non-target organisms in the seawater sample that were common to all unfiltered samples.

### Sensitivity of MiSeq Versus Ion Torrent to Reflect Differences in Relative Abundances of Target Organisms Between Treatments

**FIGURE 4:**
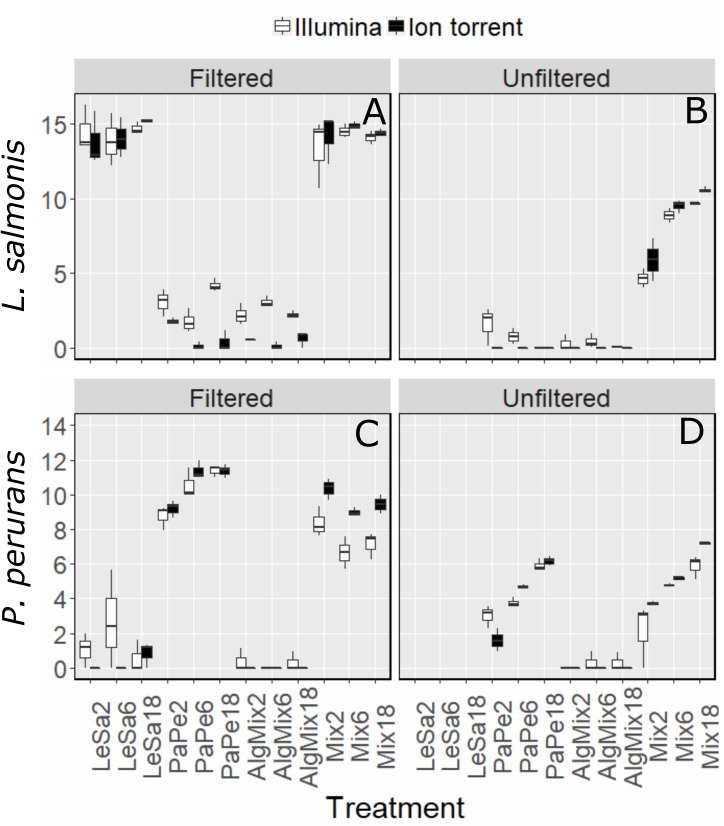
Variability in the read numbers of the two target parasite species namely *Paramoeba perurans* **(A,B)** and *Lepeophtheirus salmonis* **(C,D)** across the different treatments (see Figure 1 for treatment abbreviations). Within each treatment, the reads of each species were compared between the two sequencing methods Illumina (green bars) and Ion Torrent (gray bars) and between filtered and unfiltered marine plankton samples. For the unfiltered (not pre-filtered) water, increasing relative abundances of reads for target organisms are evident as their concentration is increased in the original sample. A similar pattern is not observed for results from prefiltered water. Low levels of cross contamination between the two target species (*P. perurans* reads in *L. salmonis* samples and vice versa) are also observed.

**FIGURE 5:**
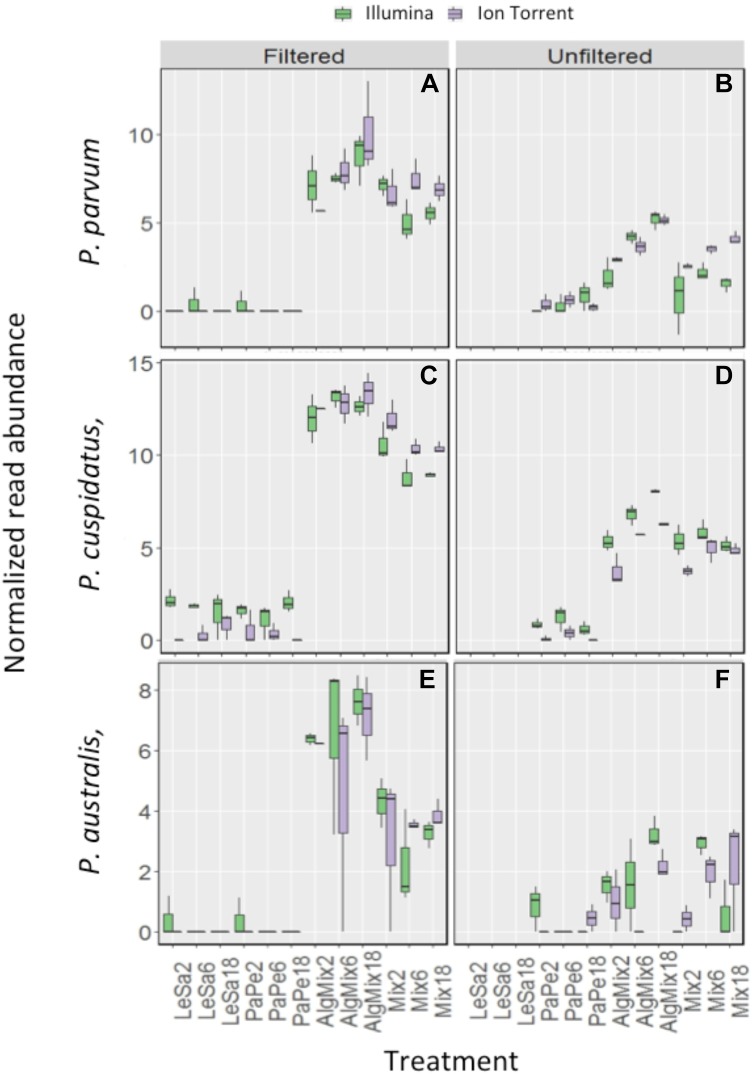
Variability in the read numbers of the three target microalgal species namely the two *Pseudo-nitzschia* species that were assigned as *P. cuspidata* and *P. australis*, but which were in fact *P. delicatissima* and *P. seriata* **(A–D)** and *Prymnesium parvum* **(E,F)** across the different treatments (see Figure 1 for treatment abbreviations). Within each treatment, the reads of each species were compared between the two sequencing methods Illumina MiSeq and Ion Torrent and between filtered and unfiltered marine plankton samples. For the non-prefiltered seawater samples, increasing relative abundances of reads for the target organisms is evident as their concentration is increased in the original sample for every organism except *P. australis*. A similar pattern is not observed in prefiltered seawater samples. Low levels of cross contamination between the target species (*P. perurans* an *L. salmonis* reads in algal samples) is also observed.

In the pre-filtered samples, the two sequencing methods were not able to reflect the different abundance levels of the two parasite species *L. salmonis* and *P. perurans* (Figures 4A,C and Table 1) and the same was true for the harmful microalgae species (Figures 5A,C,D and Table 1). In the unfiltered samples, however, the two methods were able to detect the tripling in abundance between the three abundance treatments (2, 6, and 18) for all target species apart from *P. australis* (Figures 4B,D, 5B,D,F and Table 1). Specifically, Ion Torrent was successful in 6 out of the seven sequenced treatments whereas Illumina MiSeq for 5 out of seven treatments (see Table 1). We did observe some cross contamination between samples (Figures 4, 5) – a feature more prominent on the Illumina platform than the Ion Torrrent platform.

**Table 1:** The F-ratio and corresponding confidence level (^∗^ for 95%, ^∗∗^ for 99%, and ^∗∗∗^ for 99.9% level) shows whether there is a significant difference between the 3 abundance levels for a given species within the treatment it was present.

			F-ratio and significance level	
			
Medium	Species	Treatment	Illumina MiSeq	Ion Torrent
Filtered	*L. salmonis*	LeSa	0.275	1.047
		Mix	0.537	0.368
	*P. perurans*	PaPe	11.77^∗∗^	22.51^∗∗^
		Mix	3.228	5.562^∗^
	*P. australis*	AlgMix	0.420	0.584
		Mix	2.825	0.226
	*P. cuspidata*	AlgMix	1.348	0.2861
		Mix	4.998	6.910^∗^
	*P. parvum*	AlgMix	1.354	2.119
		Mix	5.341^∗^	0.5548
Unfiltered	*L. salmonis*	LeSa	N/A	N/A
		Mix	102.67^∗∗∗^	14.818^∗∗^
	*P. perurans*	PePa	34.468^∗∗∗^	54.006^∗∗^
		Mix	8.838^∗^	526.000^∗∗∗^
	*P. australis*	AlgMix	2.924	5.799^∗^
		Mix	19.838^∗∗^	1.178
	*P. cuspidata*	AlgMix	26.257^∗∗^	18.446^∗∗^
		Mix	0.997	4.137
	*P. parvum*	AlgMix	17.783^∗∗^	24.546^∗∗^
		Mix	0.866	16.673^∗∗^


*Dark boxes indicate significant differences and also correct directionality of the effect meaning that an increase in the treatment abundance level corresponded to an increase in the reads that were sequenced.*

## Discussion

The data we present suggest that eDNA metabarcoding of the 18S SSU rDNA v9 region can sensitively and specifically identify multiple aquaculture pathogens from the complex mixture of organisms present in seawater. Furthermore, our benchmarking indicates that such detection can be achieved using a low-cost, scalable Ion Torrent sequencing platform – in some cases with better results than the Illumina gold standard. Our findings suggest that eDNA metabarcoding may represent a valuable tool in the hands of producers and regulators alike.

As our data suggest, however, several challenges remain. Taxonomic ambiguity around the assignment of the target HAB species (*P. parvum, Pseudo-nitchzia* sp.) highlights the difficulty of using short 18S rDNA amplicons to assign taxa to species level. Although the 18S V9 SSU region is thought to be useful for detecting global protist diversity (Amaral-Zettler et al., 2009), one size rarely fits all when choosing metabarcoding markers. The 18S SSU rDNA V4 region has also been shown to have high global eukaryotic discriminatory power (Hugerth et al., 2014). However, to an extent markers must be targeted at particular groups, and the 23S rDNA locus is thought to provide better discrimination for algae in particular (Yoon et al., 2016). Nonetheless the 18S v9 region we tested did provide excellent discrimination for *L. salmonis* and *P. perurans* which together account for persistent morbidity in salmonid aquaculture (Young et al., 2008; Torrissen et al., 2013; Ellis et al., 2016). In future work, it may be more appropriate to target specific organisms with tailored molecular probes, or to deploy amplicon-seq using longer read sequencing technologies to improve global species resolution. However, the former precludes the identification of novel pathogenic agents, while the latter remains too expensive to be widely adopted (Franzen et al., 2015). In addition, there is now substantial interest in adopting approaches that can be deployed “*in situ*” to allow point-of-care diagnosis. So-called “lab in a suitcase” approaches, facilitated by miniaturized and portable PCR, qPCR and sequencing (Petralia and Conoci, 2017), have made a substantial recent impact in recent biomedical contexts, such as tracking viral outbreaks (Quick et al., 2016). For salmonid aquaculture end-users, often located at remote sites far from laboratory facilities, rapid decisions are key to mitigate losses from disease outbreaks caused by agents like *P. perurans*, where early treatment is vital (Downes et al., 2015).

Although the absolute quantification of abundances in community samples remains a challenge is molecular ecology, the normalization of read data increases their accurateness in representing actual species abundances, as we have done here, so that experimentally induced differences can be more reliably reflected (Weber and Pawlowski, 2013). Nonetheless, PCR artifacts relating to runaway amplification of relatively abundant target species templates in pre-filtered water may have negatively impacted relative quantification in our study. However, in more biologically realistic samples (normal, unfiltered seawater) we were able to detect the expected increase in relative abundances in each sample according to our dilution series. Metabarcoding is poorly suited to achieving absolute abundance estimates as a direct link between the numbers of amplicons sequenced and the quantity of template in the original sample is difficult to establish – especially because two rounds of PCR are required before sequencing (Bista et al., 2018). In such cases, a single round, taxon targeted qPCR might be more appropriate (e.g., Berger and Aubin-Horth, 2018). However, PCR always carries an intrinsic risk (e.g., via differential presence of PCR inhibitors in different samples) in quantification from real-world samples (Murray et al., 2015). PCR-free approaches, metagenomic or probe-based for example, may be more appropriate for the detection of absolute biomass abundance (Bista et al., 2018).

Our benchmarking of Ion Torrent and Illumina platforms are in line with the reports of others who have done so for amplicon-seq datasets, especially in relation to higher error rates in the former (Salipante et al., 2014; D’Amore et al., 2016). However, both platforms were able to recover community compositions in our data with similar accuracy, as well as recover relative quantities of target species (D’Amore et al., 2016). The ability to run a single low output Ion-Torrent chip at a fraction of the price of the Illumina MiSeq to rapidly process only 20–40 samples, means that this technology may be more easy to adopt in a diagnostic context. Several newer Illumina models (MiniSeq, ISeq) are coming to market to address the issue of scalability. Cost may not be the only consideration, however, we were able to detect a higher rate of apparent contamination between samples from data generated using the Illumina platform (e.g., Figures 4, 5). One possible source of such contamination in our experiment is bleeding between sequence clusters, a known feature of the Illumina platform (e.g., Schnell et al., 2015), a further potential disadvantage of the Illumina sequencing approach using the exisiting MiSeq chemistry.

Our data, and that of others, does suggest that eDNA metabarcoding may soon become a useful tool for monitoring biological threats to aquaculture. Next steps could include field trials of such methodologies and corroboration with direct count data to fully validate the approach for use in the industry.

## Author Contributions

ML, SS, and KP conceived the study. LP, MD, AK, IR, and TD undertook the research and analyses. ØK-H supplied materials and reagents. LP, SS, KP, and ML wrote the paper.

## Conflict of Interest Statement

The authors declare that the research was conducted in the absence of any commercial or financial relationships that could be construed as a potential conflict of interest.

## Footnotes

1 http://www.gov.scot/Topics/marine/Fish-Shellfish/bill
