# Supplementary material for: Environmental DNA: A New Low-Cost Monitoring Tool for Pathogens in Salmonid Aquaculture
Source: Front Microbiol. 2018 Dec 7;9:3009. doi: 10.3389/fmicb.2018.03009 (PMC6292926; doi:10.3389/fmicb.2018.03009)
Supplement: Supplementary file 1 [file Data_Sheet_1.DOCX]

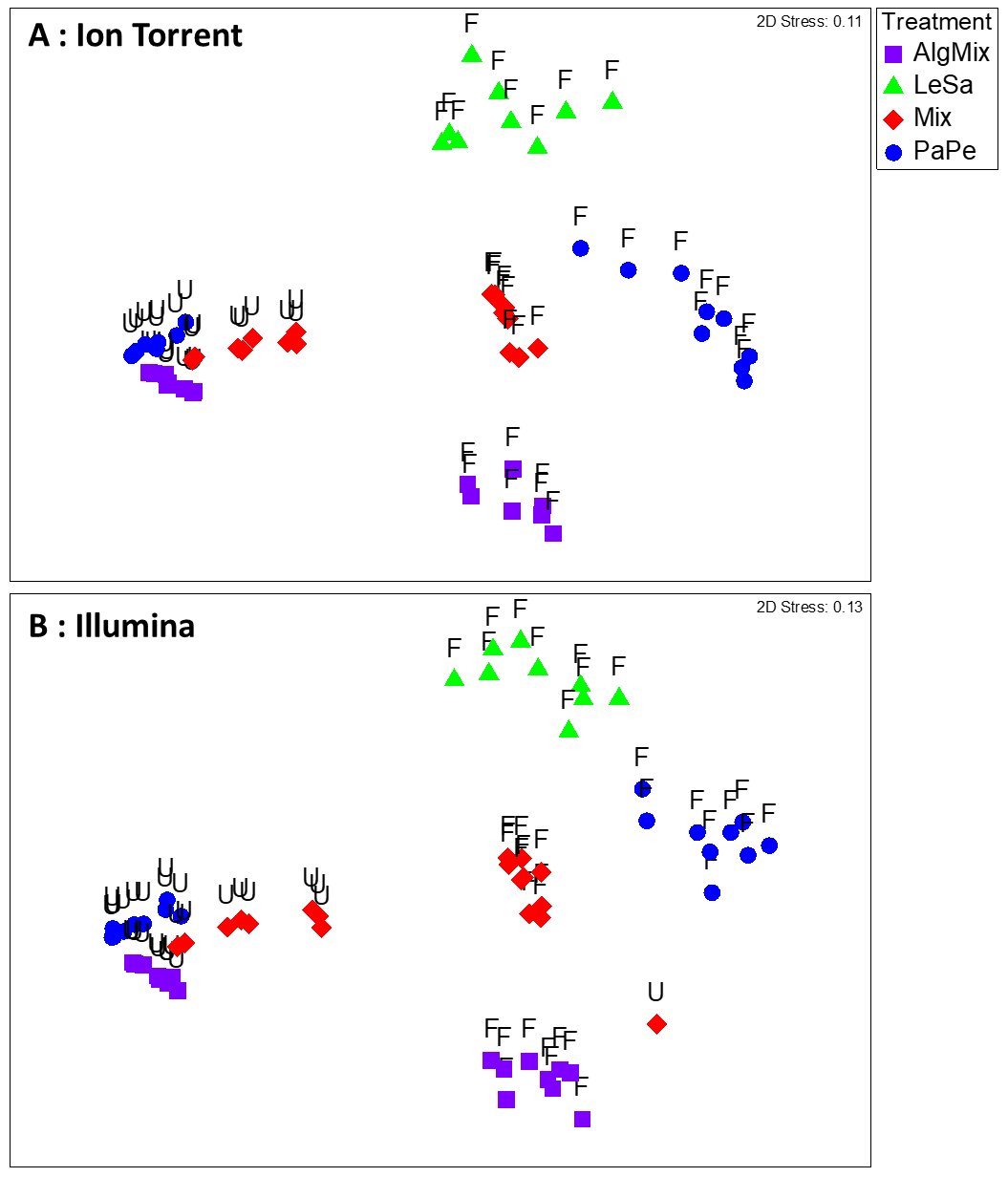


Figure S1. Multidimensional scaling ordination plots showing the similarity between samples based on their OTU-read composition. Samples are color coded for the four treatment levels: *Lepeophtheirus salmoni*s (LeSa), *Paramoeba perurans* (PaPe), algal mix (AlgMix) containing the 3 algal species, and Mix containing all the five species together. Panel A shows the pairwise sample similarity based on data derived from Ion Torrent sequencing, and panel B from Illumina MiSeq sequencing.
